# Dual career through the eyes of university student-athletes in the Republic of Kosovo

**DOI:** 10.3389/fspor.2024.1403526

**Published:** 2024-05-14

**Authors:** Masar Gjaka, Kaltrina Feka, Antonio Tessitore, Abbey Thomas, Laura Capranica

**Affiliations:** ^1^ Department of Sport and Movement Science, University for Business and Technology, Pristina, Republic of Kosovo; ^2^ Department of Applied Physiology, Health, and Clinical Sciences, Visiting Fulbright Scholar at University of North Carolina at Charlotte, Charlotte, NC, United States; ^3^ Center for Health Education and Inclusion (COHESION), Mitrovice, Republic of Kosovo; ^4^ Department of Movement, Human and Health Sciences, University of Rome “Foro Italico”, Rome, Italy; ^5^ Department of Applied Physiology, Health, and Clinical Sciences, University of North Carolina at Charlotte, Charlotte, NC, United States; ^6^ European Athlete as Student (EAS) Network, Ghaxaq, Malta

**Keywords:** sport and education, dual career, support entourage, policies, programs

## Abstract

**Introduction:**

The successful combination of a sports career and education, known as the dual career, requires cooperation and a multi-dimensional approach. Although extensive research has been conducted on dual career programs and services in developed countries, there is limited information available on the dual-career phenomenon in developing countries. This study aimed to explore the dual career experiences of university student-athletes in the Republic of Kosovo.

**Materials and methods:**

A 30-item online survey was distributed to student-athletes, addressing various aspects of the dual career. A total of 121 student-athletes (males: 63.6%; females: 36.4%) voluntarily participated in the survey.

**Results:**

These student-athletes represented 13 different sports, with 102 (84.3%) participating in team and 19 (15.7%) in individual sports. Between individual and team sports no significant differences were found regarding university and sports engagement, and the time required to travel from the university to the training venue, whereas a significant difference (*p* = 0.019) emerged for the time needed to travel from home to the training venue. Significant differences (*p* < 0.05) were found between university majors in terms of sports engagement. The present findings highlight a lack of familiarity with dual career programs among student-athletes (89.3%) and the need for dual-career policies at the university (16.5%), sport (9.9%), and national (13.2%) levels. Student-athletes faced various challenges, mainly related to limited leisure time (62.8%), academic overload (60.3%), frequent absence from classes (59.6%), and financial uncertainty (35.5%). Recommended improvements included increased financial support (66.1%), the availability of sports facilities at or near the university (48.8%), greater educational flexibility (26.4%), tutoring services at the university (25.6%), and sports clubs (19%) levels, as well as improved communication regarding existing initiatives and legal aspects (53.7% and 47.1%, respectively). The study also identified parents (98.3%), coaches (86%), and siblings (60.3%) as important sources of support for student-athletes at personal, sports, and university levels.

**Conclusions:**

In conclusion, to enhance the potential of future student-athletes in the Republic of Kosovo, relevant stakeholders in sports and higher education should collaborate closely and implement programs and services based on international best practices for dual-career support.

## Introduction

In the pursuit of their holistic development, talented and elite athletes must balance their athletic and educational endeavors, commonly known as the “dual career” (1). The combination of elite sport and education is particularly challenging (2), yet crucial in preparing and empowering student-athletes for their future societal roles after their competitive sport career has ended (3, 4). In recognizing that an elite athletic career is temporary and not all athletes can generate and capitalize sufficient income for their life course, pursuing education and acquiring professional skills will help them establish long-term career prospects, even though demanding training sessions, traveling to competitions, and engaging in various sports events puts athletes at a distinct educational disadvantage compared to their non-athletic counterparts (1, 5). These challenges have a relevant impact not only on the athlete's family and peer relationships but also on the academic paths, resulting in reduced interactions with professors/teachers, classmates, and peers, missed classes and exams, physical and psychological fatigue, and identity-related issues (6–11).

A dual career is affected by the athletes’ age, gender, motivation, sport, competitive level, university level, and academic majors, by their proximal (e.g., parents, peers, educators, coaches, and sport managers) and distal (e.g., sports clubs and federations, educational institutions) entourage, by the regional, national, and international dual career policies, and by various socio-cultural, media, and economic factors (7, 12, 13). Therefore, the quality of dual career support relies on the collaboration among multiple stakeholders, the implementation of dual career programmes and policies, a well-structured systematic monitoring system (7, 14, 15).

Primarily due to the influence of country-specific cultural and organizational regulations within the domains of sports and education, significant dual career disparities exist worldwide in the prerequisites and criteria for flexible academic paths, financial support, service-based assistance, and individualized agreements (7, 14, 16–22). Following the publication of the White paper on sports and the EU Guidelines on Dual Careers of Athletes, the European Commission endorsed the recommendation of Minimum Requirements for Dual Career Services and financed several collaborative partnerships under the ERASMUS+ programme (1, 14, 23, 24), which contributed to the development of a European dual career discourse in relation to socio-cultural context (25, 26), athlete's identity (27), competences (28), motivation of student-athletes towards sport and education (29–31), and career development and transitions of athletes (32, 33).

Despite the European recognition and the growing awareness and of the importance of dual career support for athletes, there is a limited information regarding the national standards of support and the conditions provided to student-athletes in non-European Member States. To build more cohesive communities and to support capacity building in sport activities and policies beyond Member States, recently the European Commission included in the ERASMUS+ agenda the Western Balkan area as third countries not associated to the Programme (34). In particular, the Republic of Kosovo was established in 2008 and is a multiethnic (92.9% Albanian, 1.6% Bosnian, 1.5% Serbian, 1.1% Turkish and 2.3% Roma, Ashkali, Egiptian and Gorani) country with >1.660.000 residents with an average age of 31.4 years (35). The Kosovo Olympic Committee organizes sports, with 35 registered sports federations (867 sports clubs) involving around 81,000 athletes (83% males, 17% females), and the Ministry of Culture, Youth and Sport has a limited budget for adopting programme policies according to priorities in the advancement of school, competitive, and infrastructural sports (36). At educational level, a high number of students (around 8% of the population), is enrolled in higher education institutions (8 public, 24 private), which align their education structures to the European Bologna Process to enhance the internationalization and competitiveness of the future labor force (37). However, it is necessary to ascertain the challenges that Kosovan athletes face in combining sports and education.

To ascertain the extent and consistency of dual career support of Kosovar student-athletes, the primary aim of this study was to acquire comprehensive data regarding various aspects, encompassing their: (a) socio-demographic characteristics; (b) level of involvement in sports and academics, as well as the awareness of national dual career legislation; and (c) personal, sports, and academic networks. It has been hypothesized that sport and academic engagement of Kosovar student-athletes might be influenced by the type of practiced sport as well as their academic major.

## Materials and methods

This study obtained the approval (Nr. 11796/45, date 05.10.2023). From the Institutional Review Board of the University for Business and Technology (Pristina, Kosovo). To meet the inclusion criteria for participation, individuals had to be currently enrolled in a university program (regardless of major) and involved in competitive sports at the national and/or international level. Before accessing the survey, respondents provided a written informed consent.

### The instrument

This cross-sectional study surveyed student-athletes using an online self-administered questionnaire (Google forms®). The survey was a modified version of the survey previously used by Condello et al. (19). The modifications broadened the scope of the previous survey from the university to the national level. To guarantee an equivalent translation of the original questions into Albanian language, the translation and back translation method was employed (10, 38) and changes to the survey were approved by the research group which consisted of renowned dual career experts in the field. To ensure clarity and flow, the survey was piloted and was revised based on the critical feedback of five student-athletes. Ultimately, an online survey consisting of 30 item questions was constructed, aiming to capture information regarding the major themes related to the dual career athletes such as: Socio-demographic characteristics including sex, age, university major, university level, typology of sport, competition level, previous experience at international sport events (Q1–7); sport and university engagement (Q8–12) consisting of questions related to time dedicated to sport, time needed to transfer from living site to training facilities, time dedicated to university, time needed to transfer from university site to training facilities, and challenges faced in combining sport and education; student-athletes' familiarity and awareness of dual career policies, programmes, initiatives, and available dual career documents and their sources (Q13–18); and dual career support entourage at their sport, academic and personal levels (Q19–30). Questions were closed-ended, with participants given the opportunity to provide additional elaboration as appropriate.

### Recruitment

Due to the lack of pre-existing student-athletes database, the first phase was to identify the student-athletes. Initially, the researchers contacted the public and private Higher Education Institutions (HEIs) in the Republic of Kosovo to explain the purpose of the study, and asked them to identify the student athletes in their HEIs. To increase participation, various sports clubs were also approached and informed about the study's objectives and significance. After the identification of student athletes at the HEIs and sport clubs, an email was sent to the representatives of the HEIs and sports clubs, containing detailed information about the study and a link to the survey. These representatives were asked to distribute the email to the target population of student-athletes through official communication channels. Additionally, social media and personal contacts with student-athletes were utilized to encourage maximum participation. Ultimately, the survey was distributed to 450 student-athletes of different study majors (Bachelor, Masters, and PhD) and sports, out of which 121 responded to the survey. It was emphasized that participation in the study was voluntary, and that student-athletes could withdraw at any time without providing a specific reason. To improve response rates, two reminders were planned, with a two-week interval between them. Furthermore, electronic measures were implemented to ensure that only one response could be submitted per computer, preventing multiple responses from the same individual.

### Data reduction

From the online survey, data extracted to a spreadsheet (Microsoft® Excel for Windows) were organized by the respondents' age, university major, and sport typology. Furthermore, only completed questionnaires were taken into consideration for further analysis. According to the literature (19), age (Q2) categories were 18–22, 23–27, and >27 years. Following the guidelines of the European Research Council (39), the university major (Q3) was classified into three broad categories: Social Sciences and Humanities (e.g., Business and Administration, Environmental Sciences, Finances, Foreign Languages, Law, and Psychological Sciences), Physical Sciences and Engineering (e.g., Computer Sciences, Engineering and Architecture, Integrated Design, Mechanical Engineering, Mechatronics), and Life Sciences (e.g., Medicine, and Sport Sciences/Physical Education and Sport). The sport typology (Q5) was dichotomized into team sports (e.g., basketball, football, futsal, volleyball, handball) and individual sports (e.g., artistic gymnastics, athletics, combat sports, skiing, table tennis, triathlon).

### Statistical analysis

Statistical analyzes were performed using the Statistical Package for Social Sciences version 28.0 (SPSS Inc., Chicago Illinois), with a level of significance set at *p* < 0.05. Descriptive statistics was employed to analyze the frequency of occurrence for survey questions. The responses (singular or multiple) were expressed in absolute values or percentages. For questions Q8–11, participants were presented the opportunity to provide their own values. Subsequently, the frequency of occurrence, expressed in absolute values or percentages, was computed. Specifically, this analysis considered three classes of occurrence for the time dedicated to engagement in sport and university (<11, 11–20, and >20 h). Additionally, four classes of occurrence were used to assess the time required to travel from home and university to the training venue (e.g., <30, 31–60, 61–90, and >90 min). The Kolmogorov-Smirnov test was used to verify the normality of data distribution for data from Q8–11. One-way analysis of variance (ANOVA) was used to assess the effect of independent variable (university major) on dependent variables (e.g., sport and university engagement, transfer time from home and university to training venue) with Bonferroni as post-hoc test to determine differences between groups. Additionally, the Independent Samples *T*-test was used to test the effect of sport typology (independent variables) on sport and university engagement (hr·week^−1^) and time (min^.^way^−1^) needed to travel from home and university to the training venue (dependent variables).

## Results

### Demographic characteristics (Q1–7)

**Figure 1:**
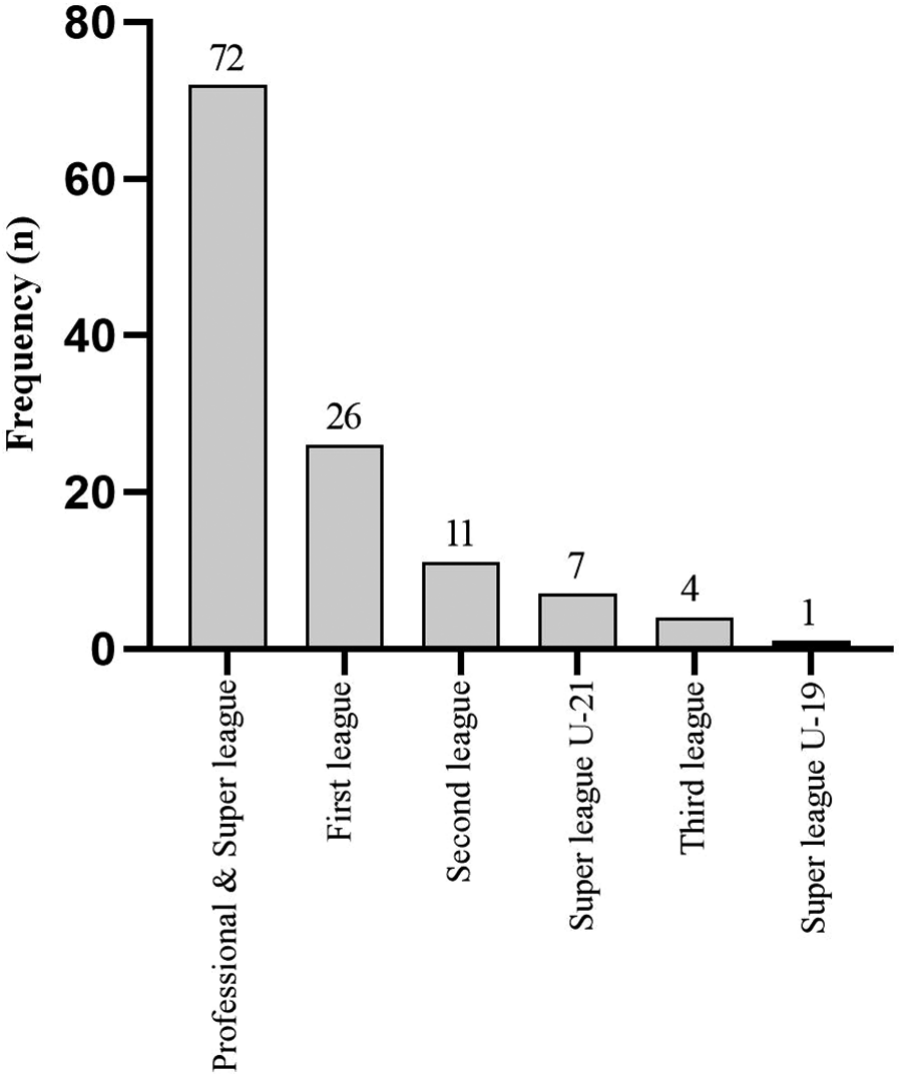
Frequency of occurrence (*n*) of respondents based on the sport level.

A total number of 121 student-athletes participated in the current study (males: *n* = 77, 63.6%; females: *n* = 44, 36.4%). Student-athletes were grouped in 6 groups based on their competitive level (Figure 1). Participants engaged in 13 different sport disciplines, with 59.5% of student-athletes competing at the professional and Super league levels. Compared to the individual sports (*n* = 19, 15.7%), the majority of respondents competed in team sports (*n* = 102, 84.3%), with football players being most represented (*n* = 66, 54.5%). 51.24% of student-athletes competed at international level, including Winter Olympic Games (*n* = 2), World Championships (*n* = 7); World Cups (*n* = 3); European Championships (*n* = 39); Balkan Championships (*n* = 25) and other international competitions (*n* = 38), whereas, no student-athletes participated in the Universiade.

**Figure 2:**
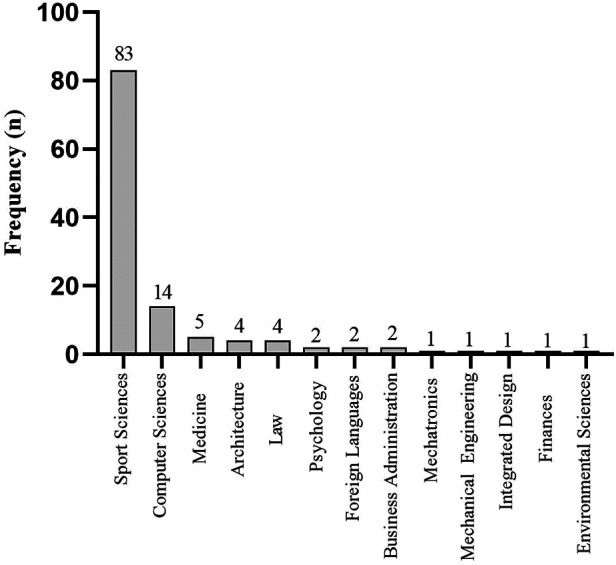
Frequency of occurrence (*n*) based on their university major.

The majority of respondents were in the 18–22 years category (*n* = 88, 72.7%), whereas those included in the older categories (23–27 years and >27 years were 23.1% (*n* = 28) and 4.1% (*n* = 5), respectively. Regarding the education level, 90% (*n* = 109) of athletes were enrolled at bachelor level and 9.9% (*n* = 12), at the master's level. For the university major, the highest number of respondents was from Life Sciences (*n* = 88, 72.7%), followed by Physical Sciences (*n* = 21, 17.4%) and Engineering and Social Sciences and Humanities (*n* = 12, 9.9%), respectively. Participants represented 13 different majors, with the majority of them (*n* = 83, 68.6%) being enrolled in Sport Science degrees (Figure 2).

### Sport and university engagement (Q8–12)

**Figure 3:**
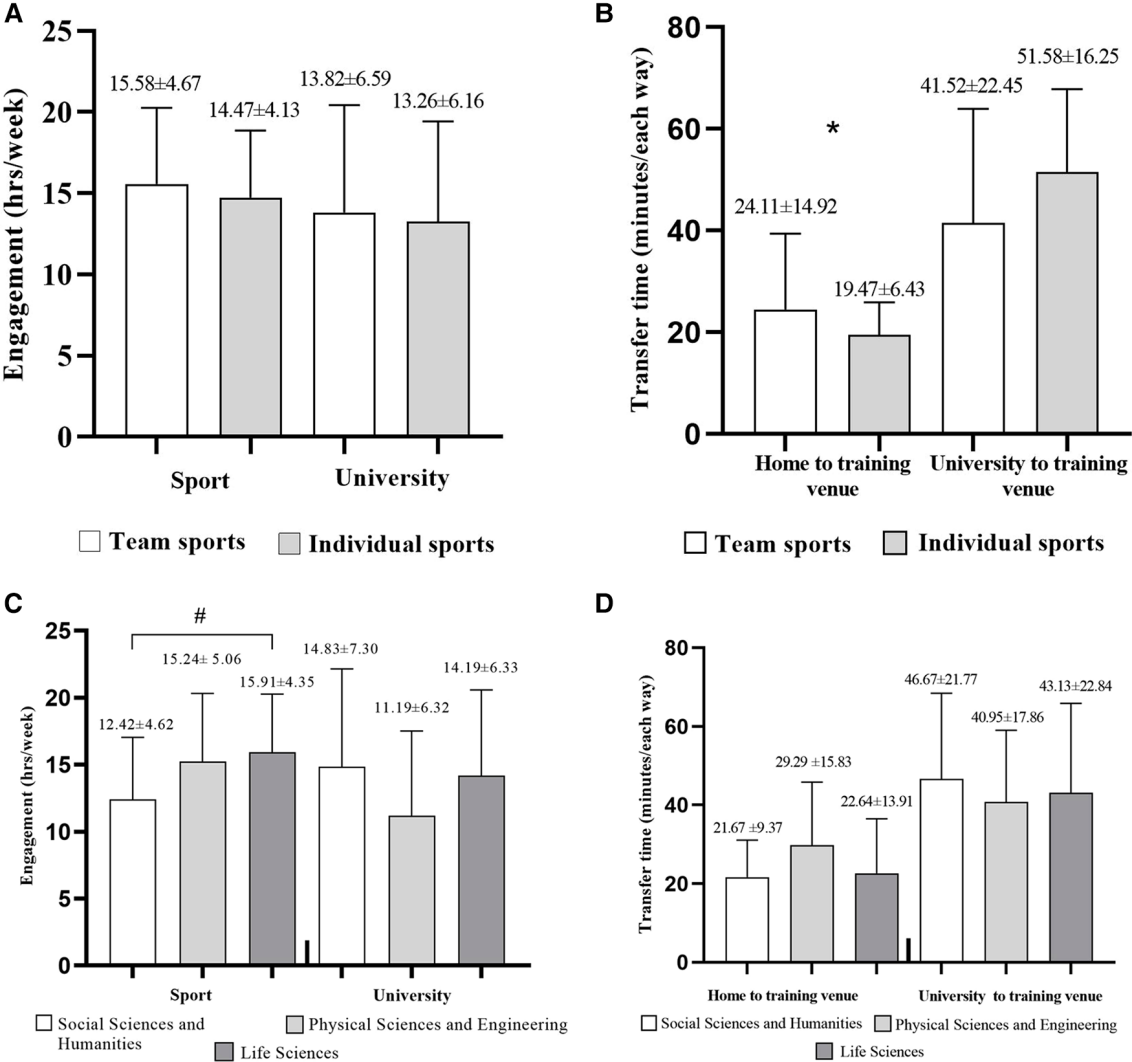
Mean and standard deviation of weekly engagement in sport and university (hours/week), and time required to transfer from home and university to training venue (minutes/each way) in relation to sport typology panel (**A,B**), and university major panel (**C,D**). *Team sport student-athletes significantly differ (*p* = 0.019) from individual sports student-athletes in terms of time needed to transfer from home to training venue. #Life Science student-athletes spend significantly more time (*p* < 0.05) in sport compared to student-athletes from the Social Sciences and Humanities major.

For sport typology, no significant differences between individual and team sports emerged with respect to academic engagement, sport engagement, and the time required from the university to the training venue (Figures 3A,B). A significant difference (*p* = 0.019) was found between team and individual sport student-athletes for the time needed to transfer from home to the training venue (Figure 3B). Moreover, a significant effect (*p* < 0.05) emerged for the university major on the time devoted to sport, with the Bonferroni post-hoc test revealing that student-athletes belonging to the Life Sciences university major spend more time (15.91 ± 4.4 h·week^−1^) compared to their Social Sciences and Humanities (12.42 ± 4.6 h·week^−1^) counterparts (Figure 3C).

Regarding the sport engagement, around 75% of respondents reported that they devote 11–20 h·week^−1^, whereas 14.9% and 10.7% declared to be engaged <11 h·week^−1^ and >20 h·week^−1^, respectively. Only 14% of student-athletes reported to be engaged at university >20 h·week^−1^, whereas 42.1% and 43.8% declaring an engagement of <11 h·week^−1^ and 11–20 h·week^−1^, respectively. Moreover, most of the student-athletes (88.4%) need <30 min to reach their training venue from home, whereas 9.9% and 1.7% spend 30–60 min and 61–90 min, respectively. Regarding the time needed to transfer from university to the training venue, 41.3% reported to need <30 min, whereas 43.8% and 14.9% declared 30–60 min and 60–90 min, respectively.

Several challenges in combining sport and education emerged, with the most prevalent being limited leisure time (62.8%), overload (60.3%), prolonged missed classes (59.6%) and absences from single classes (53.7%), missing tests and exams (35.5%), financial uncertainty (35.5%), missing training sessions due to university duties (34.7%) and extended academic path duration (21.5%).

### Student-athletes' familiarity and awareness of dual career policies, programs, initiatives, and documents availability (Q13–18)

**Figure 4:**
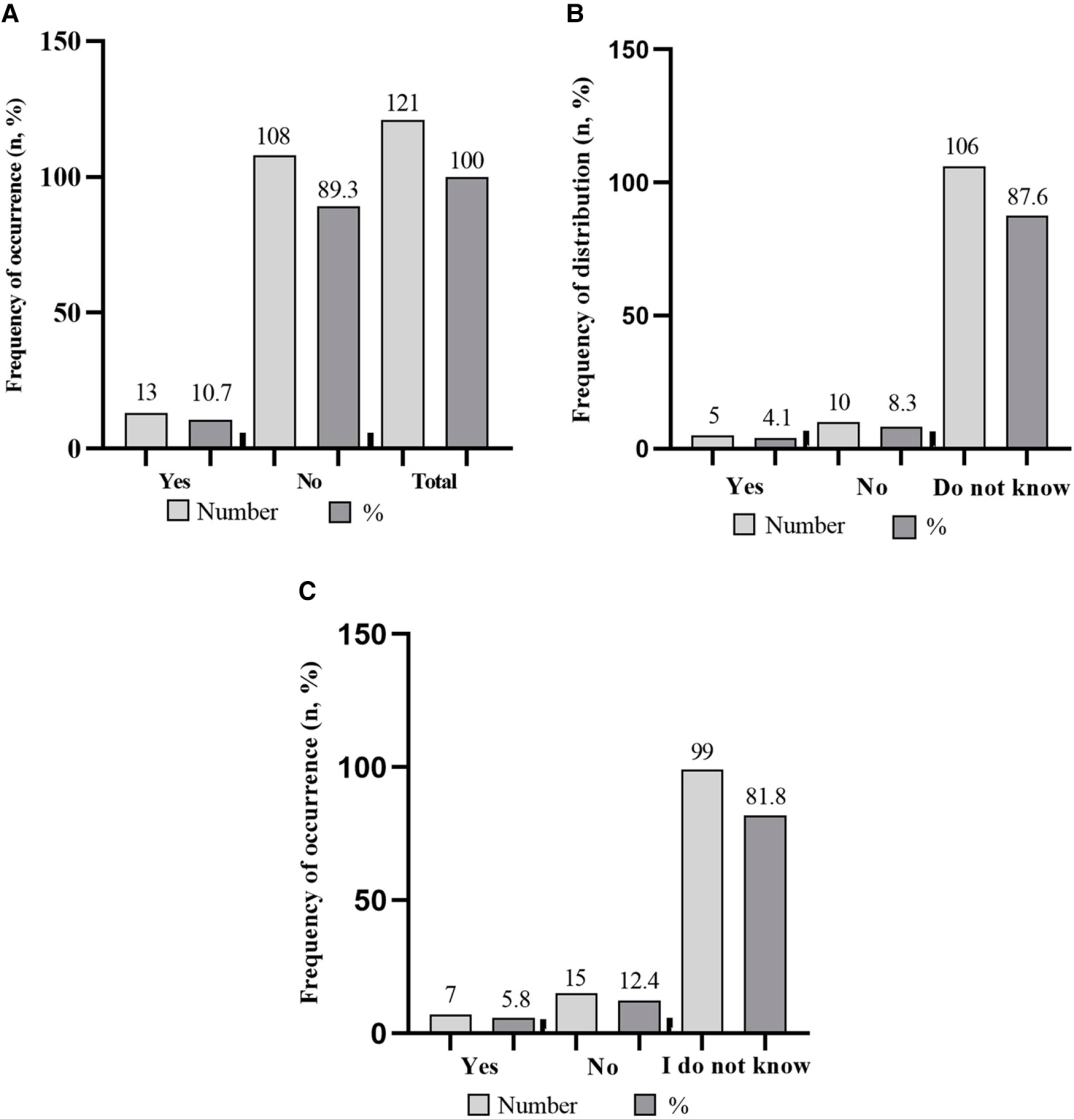
Frequency of occurrence (*n*, %) (**A**) of student-athletes stating to be (e.g., yes) or to be not (e.g., no) familiar with policies, programs or measures that facilitate the combination of elite sport and studies; (**B**) to be (e.g., yes) or to be not (e.g., no and do not know) aware of dual career policies and initiatives, and (**C**) to be (e.g., yes) or to be not (e.g., no and do not know) aware of the availability of policy documents in the field of dual career in the Republic of Kosovo.

Most of the respondents (89.3%) indicated a lack of familiarity with policies, programmes, or measures designed to facilitate the combination of elite sports and academic careers. Figures 4A–C show that respondents are generally unaware of dual career policies or initiatives (87.6%), documents outlining dual career policies (81.8%), and possible sources of information (66.1%), with internet considered a viable means of communication (33.9%). Dual-career policies ware considered an issue for university (16.5%), sport (9.9%) and governing bodies (13.2%), with the main responsibility attributed to the educational (37.2%), sport (24.8%) and government (5.8%) domains. Specifically, the regulation of the integration of elite sports and academic studies is deemed pertinent at university (41.3%) and sport federation (16.5%) levels. Regarding the involvement of public authorities in dual career policies, emphasis was placed on the national level, with 24% of respondents recognizing its significance. However, most of the respondents (76%) demonstrated a lack of awareness regarding public authorities engaged in dual career policies. Regarding the evaluation of the effectiveness of dual career policies, 43.8% of the student-athletes ignored them, whereas 26.4% stated that there is a lack of an established evaluation system, and the rest of respondents highlighting both sport and academic achievements 15.7%, only sport achievements 12.4%, and only academic achievements 3.3%, as possible means of dual-career monitoring.

### Dual career support at personal, sport and academic levels (Q19–30)

Whilst most of the student-athletes (60.3%) indicated to receive some form of support, 39.7% reported no support. Among those receiving support, educational flexibility (33.9%) was the most frequent, followed by financial assistance (16.5%) and access to sport facilities at or near the university (9.9%). Interestingly, no tutoring was available either at the university or at the sport level. Recommendations for a relevant improvement of dual career support services regarded the enhancements of financial support (66.1%), sport facilities at or near the university (48.8%), educational flexibility (26.4%), tutoring services at the university (25.6%) and sport club (19%), and better communication of existing initiatives (53.7%) and legal aspects (47.1%).

Student-athletes perceived that the majority of athletes follow a dual-career path by being engaged in sport and in higher education, with frequencies of occurrence falling within the categories of <20%, 21%–40%, 41%–60%, and 61%–80% at rates of 14%, 28.1%, 30.6%, and 19%, respectively. Moreover, 34.7% of the participants indicated a lack of awareness regarding the specific number of student-athletes benefiting from dual career support. To note, the highest estimations were associated with the frequency categories of <20%, 21%–40%, and 41–60. Whilst 9.9% of student-athletes conveyed a lack of support, 72.77% of remaining sample reported a support at the sport level and 34.71% at the academic level. At the university level, the most frequently reported forms of support included career counselling, medical support, physiotherapy and sleeping facilities. However, there was limited availability of sport facilities at/close to the university, dual career tutoring, nutritional and psychological support. In fact, 32.2% of student-athletes reported no sport at the university level whatsoever. In the sport domain, support was mainly related to sport performance, including coaching, medical support, physiotherapy, and access to sports facilities, with sports nutrition and psychological aspects reported only by 3.3% and 5.8% of respondents, respectively. Conversely, sport support did not include provision of study rooms, career counseling, and tutoring for dual career. In general, the student-athletes identified relevant dual career support entourage at the personal, sport, and university levels (Table 1). The highest frequencies of occurrence were attributed to parents (98.3%), coaches (86%), siblings (60.3%), teachers (41.3%), friends (36.4%), sport teammates (33.9%), academic staff (24%), and classmates (18.2%). In addition, university sport staff, and parent's support were reported also at the university level (10.7%, 9.9%, respectively). Although limited (2.5%), National Olympic Committee support at the university level could represent an example of integration of dual-career.

**Table 1:** Frequency of occurrence (%) of dual career supporters at personal, sport, and academic entourage levels.

Personal entourage		Sport entourage		Academic entourage	
Supporter					
Parents	98.3	Coach	86	Few professors	41.3
Siblings (sister, brother)	60.3	Sport managers	14.9	Academic staff	24
Friends	36.4	Medical doctors	24	University sport staff	10.7
Teammates	33.9	Sport psychologist	2.5	Parents	9.9
Classmates	18.2	Olympic Committee	1.7	Olympic Committee	2.5
				Administrative staff	0.8
				No one/don't know	32.2

## Discussion

In gathering information on the dual-career phenomenon through the eyes of student-athletes in the Republic of Kosovo, the main findings of the present study revealed: (a) no significant differences between individual and team sports concerning university engagement, sport engagement, and the time required to transfer from the university to the training venue; (b) significant differences between team and individual sport student-athletes on the time needed to transfer from home to the training site; (c) university major playing a significant role on the time spent in sport; (d) relatively low knowledge of dual-career policies, programmes, measures, initiatives and availability of policy documents that facilitate the combination of elite sport and studies paths. Beside the individual support provided by parents, coaches and some teachers, student-athletes urged dual career improvements at the sports, academic, and policy domains.

To meet the requirements and achieve success in both sports and academic domains, student-athletes face a simultaneous burden. Compared to the international student-athletes participating in the Universiade (19), Kosovar student-athletes spend less time in their dual career commitments, but more time in transferring from home or university site to training facilities. In considering that Kosovo is a small country, these findings substantiate the disconnection of the nation's sporting infrastructure and emphasize the importance of having sports facilities at, or near, the university. Differently from countries where sport is traditionally embedded in the educational system (e.g., USA) or a relevant part of the academic culture (e.g., UK, Australia, Canada), in Kosovo sport and education are quite separated, despite at state level the Department of Sport is located within the Ministry of Culture, Youth and Sports. In the aftermath of the war in Kosovo a systematic development of infrastructure and essential public services is still needed. The possibility to build school and university sports facilities could help future generations of youth being involved in sport and talented athletes achieving elite sport levels without the burden of long transfers (40, 41). The impacts of sport infrastructures at the academic institutions is not only at the everyday level of student-athletes but can influence the larger political and societal levels putting an emphasis on education in and through sports and promoting a dual career culture, norms and principles, as well as a cooperation between academic institutions and sports bodies.

Differently from a lack of significant effects of university major on the time spent in sports reported for international athletes (19), the present study substantiates the hypothesis that involvement in sports influences the athletes' choice of a university major (42). Specifically, when compared to their counterparts enrolled in Social Sciences and Humanities degrees, Kosovar student-athletes pursuing Life Sciences degrees dedicate more time to sports, probably due to their interest in Sports Sciences to deepen their knowledge beyond the sport practice to pursue future careers in the sport sector (43, 44).These valuable insights could foster the development of tailored programmes and initiatives that are suitable for specific contexts, sport disciplines, and educational environments (45).

Despite student-athletes should be familiar with existing dual-career policies, initiatives, and opportunities, they tend to disregard the existing legislative framework (19). Also in this study, student-athletes were unaware of European dual-career documents and their availability, indicating that sports bodies and educational institutions have to inform athletes of their rights to pursue a dual career for their holistic development. In fact, student-athletes were concerned with a lack of financial assistance, educational flexibility, adequate sport facilities at or near the university, and availability of academic and sport tutors. Conversely, many respondents reported receiving medical and physiotherapy support from the university, which is provided free of charge to all students, including student-athletes, by the National Center of Sports Medicine. At sports level, student-athletes declared a support only pertaining coaching and sports facilities, which are fundamental requisites for their athletic development. These findings indicate that dual career at educational institutions and sports bodies needs to be implemented, although the recent enforcement of the law for the development of national sports includes provisions for supporting student-athletes in terms of educational expenses (46). Additionally, the National Olympic Committee annually provides symbolic scholarships, known as Olympic Hopes, to a limited number of student-athletes. Actually, this support is not sufficient to meet the student-athletes needs of financial aid, flexibility in educational arrangements, available sports infrastructures, academic and sport tutoring, enhancement of legislation, and better communication of existing dual-career programmes and initiatives. Whilst a financial support is considered especially relevant in non-revenue-generating sports to pursue dual-career paths in a developing country like the Republic of Kosovo, the provision of tutors for student-athletes has been identified as a highly relevant and feasible dual career service (47). One positive aspect highlighted by student-athletes is the support they receive from university career centers, which need. In the Republic of Kosovo, all HEIs have their career centers that guide and advise students on career paths, but not specifically for student-athletes. As the literature emphasizes, these career centers should have trained and qualified personnel for dual career counseling (48), an area that requires improvement in the Republic of Kosovo.

It general, student-athletes naturally organize their lifestyle in relation to their sport commitment (26, 49, 50). This phenomenon has also been observed in the present study, where a significant number of student-athletes reported missing lectures and spending more time on their sports activities. Conversely, there are instances when student-athletes prioritize their academic pursuits and consequently have to sacrifice training sessions. Therefore, to optimize the effectiveness of the training-study schedule for the success of dual career paths, it is recommended to provide training centers within, or in closer proximity to, universities (51). This is particularly relevant for individual sport athletes, as team sport athletes are required to train with their teammates. Additionally, in such cases, the provision of study spaces, tutoring services at the club, and accommodations for sleeping should be considered. These initiatives would enhance the efficiency of the training-study schedule, which is vital for the successful pursuit of dual career paths (51).

Sport parenting is widely recognized for its significant impact on both sports and academics for children (52). Constructive emotional, financial, and social support from parents, along with encouragement, is crucial for motivating and achieving success for student-athletes in balancing sports and education. Conversely, negative parental attitudes can be detrimental to student-athletes, potentially leading to psychosocial challenges and dropout from sports or academics (53–55). In this study, participants emphasized the important role parents play in academic education, which aligns with the perceptions of student-athletes worldwide (19). In recognizing that the dual-career culture can benefit from a deep knowledge of the needs of parents as relevant dual-career actors, several researchers engaged in a participatory and a user-centered design approach for the development and validation of the online multilingual EMPATIA educational programme (https://edu.empatiasport.eu/eng/) tailored for parents of talented and elite athletes (9, 10, 56–58). In empowering the parents, it could be possible to manage difficult conversations and conflicts, and to envisage good and regular parents-athlete-teacher/coach communication, which is crucial to facilitate a successful dual-career alliance.

Coaches are also recognized as influential figures in shaping student-athletes, both within and beyond sports (59), as well as mentors and supporters in dual career pursuits (60). In the developmental years of athletes, coaches' mentorship role goes beyond the mastery of sport-specific skills and becomes crucial to encourage education, balanced timetables, tiredness, goal settings, and personal development for post-sporting careers (19, 49, 61–63). Therefore, sports federations need to include dual career courses in the educational and professional development programs for coaches and sports personnel. These individuals have expertise in sport-specific requirements and can assist student-athletes in managing their academic responsibilities (64).

Additionally, university sports staff and professors should be aware of the challenges faced by student-athletes and be ready to reassess their roles as facilitators in effective lifelong learning. Consequently, the recruitment of dedicated personnel as formal dual-career tutors at both the sporting and educational levels is recommended. These tutors would guide student-athletes, coaches, and professors by offering advice on personalized whole-life development plans and monitoring progress (14, 48). In this respect, the online European Dual Career Toolkit (https://starting11.eu) could assists institutions in sport, education and the labor market in implementing effective dual career services for athletes. Furthermore, to organize a dual career process based on objectives, content, control, and evaluation systems, HEI decision-makers, rectors, and sport faculties could consider the methodological approach and the summarized experiences of the European More Than Gold Guidelines and Manual (65, 66).

Overall, the present exploratory study on the Kosovar student-athletes' perception of their dual career challenges resulted in line with the quest for implementation of dual career policies and provisions reported for different European Member States (56). In considering the infancy level of its dual career, the present findings set the foundation for the development of the dual career in the Republic of Kosovo. To build impactful and sustainable development of dual career policies and provisions, the Republic of Kosovo could profit from the accumulated valuable knowledge and experiences of the European dual career discourse. Looking ahead, dual career in the Republic of Kosovo could be shaped in three medium- and long-term interrelated factors. First, the recognition of the athletes' right of a holistic development and the crucial importance of student-athletes as prepared future actors of the society, being also role models for the next generations. Second, the need of a substantial alliance between the sport and academic environments for building dual career agreements, regulations, and infrastructures for implementing and administrating dual career services. Third, the need to educate dual career actors (e.g., parents, coaches, teachers, sports managers, teachers, and academic staff) to build a strong supportive entourage for empowering athletes to pursue their dual careers.

The current study has some limitations which are worth highlighting. First, it should be acknowledged that the response rate for this study was relatively low. Furthermore, the uneven recruitment of student-athletes across sport typologies, and university majors could potentially constrain the generalizability of the research findings. However, to attain a more comprehensive understanding of the interplay between education and sports, it is imperative to conduct extensive research involving a larger sample size of student-athletes with diverse backgrounds in terms of both study majors and types of sports.

## Conclusions

Based on the findings of the current results, it can be concluded that the establishment of the minimum standard of dual career services at national level is highly recommended in the Republic of Kosovo. Additionally, responsible bodies are encouraged to offer specific educational programs for dual career service providers at academic and sport levels, including parents, coaches and university staff, who play a strong supporting role in the pursue of dual career by student-athletes. Furthermore, it is essential to the success of the student-athlete, that academic institutions and sports organizations, such as sport clubs, to collaborate closely to create a supportive environment for student-athletes. This collaboration should involve the implementation of specific accommodations, such as flexible academic and sports timetables, online learning opportunities, tutoring services, studying spaces at the club, and support for student welfare. The implementation of these measures is critical in helping student-athletes manage their time effectively and overcome challenges related to balancing their dual career paths (7, 14). Moreover, seeing the lack of awareness the student-athletes have regarding their dual career rights, policies, programs, services, financial resources, and logistic support in place, dual-career stakeholders are strongly encouraged to use the right promotion means to inform student-athletes regarding these aspects.

## Data Availability

The raw data supporting the conclusions of this article will be made available by the authors, without undue reservation.
